# “People like numbers”: a descriptive study of cognitive assessment methods in clinical practice for Aboriginal Australians in the Northern Territory

**DOI:** 10.1186/1471-244X-13-42

**Published:** 2013-01-31

**Authors:** Kylie M Dingwall, Jennifer Pinkerton, Melissa A Lindeman

**Affiliations:** 1 Menzies School of Health Research, Institute of Advanced Studies, Charles Darwin University, Darwin, NT, Australia; 2 Centre for Remote Health, Flinders University, Alice Springs, Australia; 3 Menzies School of Health Research, PO Box 4066, Alice Springs, 0870, Australia

**Keywords:** Cognition, Assessment, Cross-cultural, Testing, Indigenous, Aboriginal

## Abstract

**Background:**

Achieving culturally fair assessments of cognitive functioning for Aboriginal people is difficult due to a scarcity of appropriately validated tools for use with this group. As a result, some Aboriginal people with cognitive impairments may lack fair and equitable access to services. The objective of this study was to examine current clinical practice in the Northern Territory regarding cognitive assessment for Aboriginal people thereby providing some guidance for clinicians new to this practice setting.

**Method:**

Qualitative enquiry was used to describe practice context, reasons for assessment, and current practices in assessing cognition for Aboriginal Australians. Semi-structured interviews were conducted with 22 clinicians working with Aboriginal clients in central and northern Australia. Results pertaining to assessment methods are reported.

**Results:**

A range of standardised tests were utilised with little consistency across clinical practice. Nevertheless, it was recognised that such tests bear severe limitations, requiring some modification and significant caution in their interpretation. Clinicians relied heavily on informal assessment or observations, contextual information and clinical judgement.

**Conclusions:**

Cognitive tests developed specifically for Aboriginal people are urgently needed. In the absence of appropriate, validated tests, clinicians have relied on and modified a range of standardised and informal assessments, whilst recognising the severe limitations of these. Past clinical training has not prepared clinicians adequately for assessing Aboriginal clients, and experience and clinical judgment were considered crucial for fair interpretation of test scores. Interpretation guidelines may assist inexperienced clinicians to consider whether they are achieving fair assessments of cognition for Aboriginal clients.

## Background

Aboriginal Australians in the Northern Territory (NT) are often exposed to a number of cognitive risk factors including poor nutrition, infections, head injury, violence, parental and personal substance abuse, and complex trauma associated with disadvantaged social circumstances [1,2]. Subtle cognitive impairment can be difficult to detect through clinical interview or normal conversation, and the ability of clinicians to accurately estimate cognitive functioning without the use of standardised tests can be poor [3]. However, existing cognitive tests can rely heavily on the use of the English language, require written responses and resemble mainstream educational processes [4]. Poor English literacy, a lack of formal education, as well as differing concepts of numbers, time and space can mean that Aboriginal people may have limited experience with the knowledge base from which such tests are derived [5-11]. Furthermore, test interpretation often relies upon inappropriate normative data, typically drawn from non-Indigenous samples [12]. Consequently, for Aboriginal people cognitive dysfunction often remains undetected, misdiagnosed and ultimately, untreated.

Inability to identify cognitive impairment reliably for Aboriginal people may place them at further disadvantage. For example, cognitive impairment may reduce an individual’s insight into their own behaviours and their desire or ability to seek or respond to available treatment [13,14]. Furthermore, when in contact with the criminal justice system, being both Aboriginal and having a cognitive disability may be a “dual disadvantage” [15; p. iii]. Such individuals may be less likely to know their rights when questioned by police, less able to assist in their defence, be at risk of victimisation in custody, and at risk of reoffending [15]. Aboriginal people may therefore experience detention as a result of inadequate assessment, treatment and services [16].

While the inadequacy of mainstream service provision and cognitive assessment for Aboriginal Australians has been recognised for a number of years, little practical development has occurred [17]. An extensive review of the literature critiquing the cognitive tests currently and previously used with Aboriginal Australians, found very few tools that were appropriately developed or validated for this group [4]. Regardless, anecdotal evidence suggests that alternate tests are being utilised in different ways within clinical practice. However, there is no documentary evidence detailing what is being used and how.

Clinicians new to the field of Aboriginal health and those wanting to perform evidence based practice therefore have little guidance when it comes to assessing cognition appropriately for Aboriginal clients. As a result, some Aboriginal people with cognitive impairment may lack fair and equitable access to services. This project aimed to address this knowledge gap by drawing on the expertise of clinicians in the field. In doing so, it sought to bring an expanded evidence base to the conduct of cognitive assessments in practice and to identify how assessments are being conducted to achieve culturally fair measures of functioning.

## Methods

This study sought to describe current clinical practice in assessing cognition for Aboriginal Australians using qualitative enquiry. Purposive sampling with snowballing was used to recruit participants. Participants were professionals who had conducted cognitive assessments for Aboriginal people, including 11 general psychologists (including one Aboriginal psychologist), three school psychologists, one provisional psychologist, three occupational therapists (OTs), one speech pathologist, two aged care nurses, and one physician (N = 22). Clinicians had a range of experience working with Aboriginal Australians, from few to over 20 years experience. A participant reference group consisting of some participants, and an additional OT and Aboriginal researcher was established, meeting at three time points, to guide the research process. The project was also reviewed by a Menzies School of Health Research (Menzies) Indigenous Reference Group for advice and guidance. Ethical approval was granted by the Human Research Ethics Committee for the NT Department of Health and Menzies (including an Aboriginal sub-committee) and the Central Australian Human Research Ethics Committee.

Written informed consent was obtained prior to conducting 18 individual or small group (2–3 people) semi-structured interviews with participants either in person or over the telephone. The interviews took 45–60 minutes and consisted of the same stem questions used for all participants. Questions enquired about the practice context, reasons for assessment, current assessment methods, outcomes of assessments, and strategies for managing identified impairments. Rich data were collected, but due to publication constraints, only selected themes as they relate to assessment methods are reported here. Additional data will be published in subsequent papers. Interviews were audio-recorded and transcribed verbatim. Transcripts were critically analysed using thematic analysis assisted through NVivo software[18]. Common themes were generated by organising interview data into a system of coded patterns, concepts and categories. Themes were identified by author one, independently cross-checked with co-authors then discussed until consensus was reached. During the data collection and analysis phase, emerging themes were presented on two occasions to the participant reference group for verification and discussion. Pseudonyms are used to present results.

## Results

### Reason for and importance of assessment

Most practitioners agreed that a good clinical justification for a cognitive assessment is needed as assessment can have important and long-term consequences.

*“Once it’s written in a file, it’s there forever.”* (Victoria)

*“You need to really sit down with the referrer and say ‘why do you want this done?’ ‘What purpose is it going to be put to?’”* (Chantelle)

Clinicians would generally conduct cognitive assessments for either therapeutic or bureaucratic reasons, sometimes both.

*“The… testing side of things provides information for funding purposes [and] it provides information for developing educational adjustment plans for students.”* (Sarah)

Therapeutic reasons generally revolved around informing the intervention. This included identifying the impact of impairment on daily functioning, identifying strengths to work with, tailoring treatment to the capabilities of the client, assessing how well an individual might cope in a specific program (i.e. program fit), developing educational support interventions, and providing support to others (e.g. family, teachers) in assisting the client.

*“I think you have to do some sort of assessment because you need to. As in to be able to offer services and support. And also to try and support or assist teachers in gaining the best from the kids.”* (Sarah)

*“Assessment is to determine eligibility for the program, and if they are not suitable for a group program, then what interventions would be suitable.”* (Hayley)

Working from a strengths approach, rather than a deficits approach featured strongly.

*“If [the child] looks dull but they’re not dull, you want to be able to pick it up…In my view the power of [the tests] isn’t to say this kid’s dull, it’s to say he’s bright here and he’s bright there, [and] to get the teachers and the adults to be more optimistic about the child.”* (Martin)

Another important therapeutic reason for conducting a structured assessment included teasing out the issues based on assessment not assumptions, thereby minimising reliance on subjective opinion which itself may contain cultural bias.

*“Getting an idea of… [whether] there are deficits, [and] where the deficits [lie] and [do] they match up with the typical deficits of [someone with] alcohol injury or is there a different … pattern there… that we’d be better off to… address?…Rather than just a blanket [assumption that] ‘this person is Indigenous, they live in the river, they must be alcohol’. No, pull back and assess.”* (Julie)

Bureaucratic reasons included legal/court requirements, to assess culpability, fitness to stand trial or victims’ compensation claims; employment reasons, to identify those who should be on pensions; adult guardianship assessments, to identify those who require guardianship orders; resource allocation issues, to identify those who qualify for additional support; service access/gate keeping reasons, to identify whether the client can access a specific service, and as support for funding bids in a particular sector or region. Bureaucratic reasons generally required an assessment via standardised assessments, with more importance placed on the test score.

*“People like numbers.”* (Scott)

*“Some of the bureaucracy is organised in that direction, they want assessments.”* (Martin)

The reason for the assessment was considered in selecting the assessment tool. Emphasis was placed on tailoring the assessment to the individual according to suspected reasons for impairment, language ability, education, age, acculturation or other factors.

*“Yeah, you use different methods for different people really.” (*Michelle)

### Assessment methods

A range of mainstream standardised assessments were discussed and clinicians stressed the importance of tailoring an assessment to individual clients.

*“I'd …be loathe to pick a couple [of tests] out just because I wouldn’t want people to say ‘just do this sub-test and go’. It depends really too, some Indigenous people really have quite a Western life, they’ve got post-grad education, they’ve got vocabulary, language … What we would be wanting to do is match it to the person.”* (Felicity)

Even experienced clinicians, working with Aboriginal people for over 15 years, did not consider themselves experts, and stressed that there are no hard and fast rules.

*“[I’m] experienced but always learning. I mean, I just think there is so much more to understand and know about assessing cognition with Indigenous kids.”* (Sarah)

It was considered necessary however to ‘know’ the test well in order to be able to interpret it appropriately.

Table 1 presents a list of assessments used by different professions in different contexts. The assessments used most often are discussed below.

**Table 1:** Assessment use reported by discipline and context

	**General**	**Research**	**School**	**Prison**	**Aged & disability**	**Hospital**			
**Assessment tool**	**Psych**	**Psych**	**Psych**	**Psych**	**Nurses**	**OT**	**Speech path**	**Dr**	**Total**
Kimberly Indigenous Cognitive Assessment (KICA)	2			1	2	3	1	1	**10**
**Matrices**									
Ravens Progressive Matrices		1	3	4					**8**
Kaufman Brief Intelligence Test (KBIT)	3			2					**5**
Naglieri Nonverbal Ability Test (NNAT)	1		3						**4**
Test of Non-verbal Intelligence (TONI)	1								**1**
**Wechsler Scales**									
WAIS/WISC	3		1	2					**6**
Wechsler Non Verbal			2						**2**
Wechsler Memory	2			1					**3**
**Adaptive Behaviour Scales**									
Adaptive Behaviour Assessment System			2	1					**3**
Vineland Adaptive Behaviour Scales	1		1	1					**3**
BRIEF				1					**1**
Achenbach Behaviour Checklist	1								**1**
Bender-Gestalt II			3						**3**
Mini. Mental State Examination	2			1					**3**
Addenbrooke’s Cognitive Examination	1							1	**2**
Genograms	2								**2**
Rey Complex Figure Test	1	1							**2**
Rowland Universal Dementia Assess. Scale(RUDAS)	2								**2**
Schonell Reading Test		1		1					**2**
Activities of Daily Living (ADLs)				1					**1**
Functional Communication Assessment							1		**1**
Functional Impairment Measure (FIM)							1		**1**
Boston Naming Test							1		**1**
Comprehensive Aphasia Test							1		**1**
Junior A&B		1							**1**
L'hermitte Signoret		1							**1**
Otis-Lennon School Ability Test		1							**1**
Peabody Picture Vocab. Test				1					**1**
Quick Neurological Screening Test			1						**1**
Stanford Binet IV			1						**1**
Western Aphasia Battery							1		**1**
Westmead PTA						1			**1**
WHIPPSI	1								**1**
Porteus Maze		1							**1**

*Note Psych* psychologist, *OT* Occupational therapist, *Speech Path* Speech pathologist, *Dr* Medical doctor.

#### Kimberley indigenous cognitive assessment (KICA)

The KICA [9,19,20] is a dementia screen that assesses orientation, free and cued recall, language, verbal fluency, copying sequence pattern and ideational praxis and was recognised as one of few cognitive assessments appropriate and developed for Aboriginal people. As a result, it was sometimes utilised for purposes other than assessing dementia. This was recognised as a significant problem for some clinicians. It was considered inappropriate for younger people or non-dementia related referrals because it had not been normed for those under 45 years and does not necessarily test the right functions for intellectual disability or substance misuse injury, for example.

*“And I know parts of it will fit into both needs but it’s not appropriate to use a tool for dementia for assessing whether someone is intoxicated and can make safe decisions.”* (Natasha)

However other clinicians were not so strict, and suggested the KICA was useful as a “loose guideline” or an “ice breaker” among younger or non-dementing clients and qualitative information could be gleaned, to inform the overall clinical impression.

*“If someone fails the KICA and they're too young… it gives you a guide as to what [other test] to use, [and] you look at how they failed it.”* (Felicity)

While there were mixed opinions about the KICA, most were positive, if used for its intended purpose.

*“It really is a good assessment for dementia screening. It is a wonderful assessment. It is just that its use has been exploited a little bit. It’s seen as the answer to everything.”* (Victoria)

Particularly liked was the KICA guide for involving family and collecting contextual information. The training, the guidelines for assessing people with low vision and it being freely available were also rated positively. However, the comprehensiveness of the contextual component presented some issues regarding the time taken to complete and inability to hold the clients’ attention for prolonged periods.

*“I really like doing it but it’s so time consuming.”* (Victoria)

There was however, a general acknowledgement that all assessments have their limitations, even Aboriginal specific ones, reflecting the fact that the assessment process itself is based on different cultural concepts.

*“Timed response doesn’t work and ‘look at these pictures and later on I’m going to ask you’ and then, ‘so remember what those pictures were?’ But [the client] will talk about lots of other things and… it just doesn’t…work and it’s not a cognition thing… I just don’t think testing in that manner works, or [even] wanting a response in that manner, they sort of seem to think that you’re a bit crazy.”* (Lisa)

#### Wechsler scales

The Wechsler intelligence scales were used exclusively by psychologists as they were familiar with them, mainstream systems relied upon them and there was a perceived relative lack of alternatives.

*“Well, we’re stuck with the sub-sections of the WAIS, and so forth, and you’ve got to choose the right ones…Wechsler scales, that’s what I know best. I’ve used them for so long and I can modify [them]. I know, looking at the numbers, I know what it means.”* (Sampson)

*“…at the end of the day, our systems function on the score of the WAIS; even though you want to record it responsibly in terms of cultural bias.”* (Felicity)

Psychologists who utilised these scales used predominantly performance or non-verbal subtests of the general intelligence scales (i.e. Wechsler Adult Intelligence Scale (WAIS) & Wechsler Intelligence Scale for Children (WISC)), or the Wechsler Memory Scale (WMS) that typically assesses functions such as perceptual reasoning and problem solving, processing speed and memory. Interestingly only the school psychologists utilised the Wechsler Non-Verbal (WNV), but it was perceived by Belinda as a very “peculiar” test (due to some of the pictures) with a sense of “unevenness” to it. The full scale IQ score of the WAIS was generally not considered valid and selection and interpretation of subtests relied heavily upon experience and clinical judgement. For example, an individual’s level of English, education and acculturation were considered when selecting Wechsler scales, often conceptualised as a difference between ‘remote’ and ‘urban’ based clients. One well regarded psychologist who had been assessing Aboriginal people for over 20 years used specific subtests, albeit with certain caveats. For example, the coding subtest is timed and “Aboriginal people don’t really get into being timed, so you’ve got to be careful how you interpret the results” (Martin).

*“In the WAIS I’d use the blocks, I’d use the coding I’d use the digit span and I might use comprehension if their English is good enough…I’d use memory for designs…now that’s meant to be sensitive to alcohol impairment…Memory for designs, ten second exposure is useful, you can carry the cards in your pocket, that’s another reason why it’s useful and it’s not traumatic for them… I use the digit span verbal… with Aboriginal people… but they don’t usually like it very much. The other one I use out of the Wechsler is called … visual memory span…where you have a card with dots on it and you touch the dots in a certain sequence and they’ve got to touch the dots in the same sequence, so there’s no language, once you’ve got the trick across. So you do them forward and then you get them to do it in reverse and they often do the reverse better than [they do] the first.”* (Martin)

Many agreed that there is cultural bias in many of the Wechsler scales particularly due to language and education differences and most agreed that the WAIS full scale IQ score is invalid.

*“So you might know that no, they don’t have an intellectual disability even though [the score] said they do, but they [actually] don’t. But what is true about it is that they will operate at that level within that Western education system”* (Belinda)

*“Ethically the WAIS is inappropriate to use in an Indigenous environment.”* (Kirsty)

#### Matrices (including KBIT, Raven’s, TONI, NNAT)

*“When I went back and looked at their WAIS scores, their best subtests were the matrices.”* (Kirsty)

Many psychologists (including an Aboriginal psychologist) found the matrices components of tests such as the Kaufman Brief Intelligence Test (KBIT), the Raven’s Matrices, the Test of Non-verbal Intelligence (TONI) and the Naglieri Non-verbal Ability Test (NNAT) to be useful and completed well, even enjoyed by Aboriginal people. These tests measure reasoning ability and were thought to be intuitive, quick and easy to administer and require little reliance on language.

*“I would use the Ravens; that would be my weapon of first choice in all this… It’s quite intuitive in many ways… So I have a degree of confidence that it is probably relatively culturally unbiased. So yeah I have a degree of faith in that. And others have also said similar.”* (Scott)

The KBIT was seen by some as the most engaging as they included pictures of people etc., rather than using purely geometric patterns, but were again used with caution.

*“That’s why I like the KBIT, because it is relatively quick, you get really quite an impressively reliable score, even though you would still make allowances – you wouldn’t take the literal score… you wouldn’t pay attention to that, but you’d pay attention relative to those other little tests, because almost certainly it’ll be an underestimate of the person’s real ability because of acculturation.”* (Martin)

*“The TONI was very boring in the way it was presented; and very repetitive just in its formatting.”* (Sarah)

The TONI was used least out of the matrices tests, but highly regarded by one psychologist who was very practiced in its use.

*“I like the TONI and the Rey [Complex Figure Test]. I’ve just grown very fond of them.”* (Kirsty)

This psychologist reported “on average, those who did the WAIS and then the TONI would score 10–15 points lower on the WAIS than the TONI”.

The NNAT was used predominately by the school psychologists, given that it is a test for children.

#### Observation, informal, or functional assessments

Almost all clinicians would supplement their standardised assessments with qualitative or informal observations and tasks during the clinical interview. They might ask clients to complete certain tasks along with monitoring specific behaviours or presenting state.

*“In the course of providing therapy, I would informally be assessing these sorts of cognitive functions. Just as a matter of course, that would be informal.”* (Neil)

*“How alert are they…have they been up all night, or are they stoned? …and usually to get a bit of an idea of memory, I always give them my name and ask them again later. I say ‘I’m going to ask you that later’.”* (Martin)

Other things observed or assessed informally included speech patterns, perception (e.g. hearing, vision), social interactions, memory for biographical data, identifying and remembering objects, money management, attention and distractibility, alertness or activities of daily living. For example, some allied health professionals would ask a client to make a cup of tea and observe for sequencing, identifying what items are required etc. Some would observe the client ordering off a takeaway menu asking questions for example, ‘How much money would you give?’ and ‘Would you get any change back?’ They might also ask clients to name objects in the room, identify an object’s use, and ask them to recall those objects later. Some psychologists would use playing cards and ask clients to sort the cards into suits or sum the values of two cards.

*“I might put out an ace and a ten and say ‘How much is that?’ … I ask them what they play and how they play it…And a lot of them are quite good at it. But if they don’t know anything about cards then that means nothing, but if they say they play, and they can’t do this simple arithmetic, you’ve got to say, ‘What’s going on here?’”* (Martin)

*“Specific language use is quite interesting, and if information is repetitive and things like that. With skills and watching people operate through a normal daily environment it's quite enlightening to watch the standard procedural skills, but it's also very useful to see when they're interrupted. So how someone responds to an unexpected circumstance, forcing them off their standard behavioural script, and they … improvise a response… looking at how effective that response is, [and] how long it takes them to actually develop an alternative response. That information can be really insightful.”* (David)

**Figure 1:**
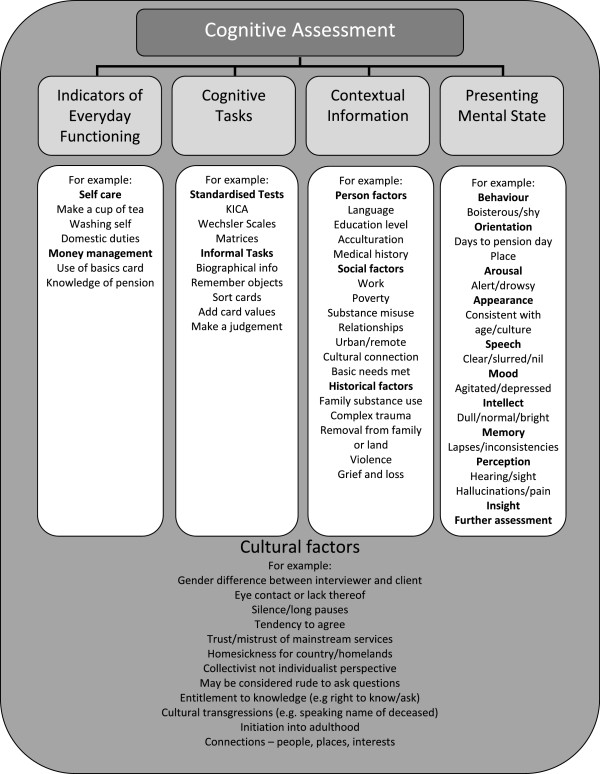
Schematic of assessment process with examples.

Generally clinicians reported it was essential to use more than one assessment and more than one form of data collection and cultural factors impact all of these (see Figure 1).

*“I would never, ever do one. Never, ever, ever. I normally do three.”* (Belinda)

### Conducting the assessment

#### Building rapport

Clinicians stressed the importance of engaging the client, and building rapport was usually the clinicians’ first priority.

*“The first step is developing rapport and often the Indigenous worker will meet them first and then they’ll have to vouch for us. So there’s no way known we can just sit them down and say, ‘Okay, now we’re doing the assessment.’”* (Chantelle)

Rapport-building might include conducting the assessment in a comfortable setting for the client, which may be away from the office or commonly, outdoors.

It’s about making the individual feel comfortable and there is also often a degree of personal disclosure on the clinician’s part in order to find some kind of connection with the person.

*“Oh yeah, there’s that level of personal disclosure, I’ll talk about my family background and what my connections are, I’ll talk about football, I’ll talk about fishing or whatever is going to start a connection between us.”* (Scott)

*“If you appeal to the sense of humour, that’s very good.”* (Sampson)

It often takes a lot of time to build rapport, with some clinicians reporting the importance of returning many times until a level of trust is built.

*“Most people would go twice; we go ten times or twenty times or whatever. And you just keep going until you sort of feel that you know them more and they trust you more and they’ll talk to you more and they’ll give you more information.”* (Lisa)

#### Aboriginal liaison or interpreter services

Clinicians advocated for the use of Aboriginal Liaison Officers (ALOs) and interpreters in conducting assessments. This was generally seen by all as good practice.

*“We … always have an ALO with us. I won’t do [the assessment] without an ALO present… And people, even if they speak good English, will say things to Aboriginal people that they won’t say to us.”* (Victoria)

ALO’s tended to be used to assist with rapport and cultural understanding (and many organisations employ ALOs for this purpose), whereas interpreters have a more specific role with translation of language, and need to be engaged through an interpreter service or found privately through local contacts. The use of interpreters was acknowledged as difficult and not always possible given the setting, timeframes and availability of appropriate people. Time was spent coaching the interpreter in terms of what to say and what not to say when conducting an assessment. A further issue reported arose when an interpreter was not able to translate words such as ‘left’ and ‘right’, advising the clinician that the words did not exist in their language. As the clinician’s previous experience of this language did not concur, another speaker of the language was consulted, confirming that the words did exist. The interpreter did not appear to have the same depth of understanding of the language leaving the clinician uncertain about the level of reliance to place on interpreters (reference group meeting discussion).

#### Modification or adaption

When asked if they conducted the assessment in the standardised way or with some flexibility, most clinicians reported that they would pretty much “stick to the script”. However, there were moments of flexibility around that in terms of introducing what was going to occur, starting with the easier (lower age) items to give clients a chance to practice the task, or using language as well as gesture in instructions.

*“I try to do [the tests] as standardised as possible, but put them in an accessible way. So I tend to introduce what it is that I’m doing, why I’m doing it that way, [explain] that ‘We will be proceeding through things in a sequential way, one after the other, we would be going from one thing, I might be asking a question or I might be asking you to draw something’ and so on. And then I’ll say ‘Now we come to this part’. So I’ll try to outline it, but that’s the only way I really change it. If I need to simplify the language, then I will or the interpreter might need to do whatever needs to be done.”* (Neil)

Whilst assessments would generally be delivered according to the standardised instructions, modifications to wording or particular item content were sometimes made in order to make the test material more relevant or localised to the immediate context. For example, items requiring memory for addresses were changed to reflect Northern Territory addresses, naming the prime minister of Australia rather than the president of the country where the test was developed.

*“This other one, with the pictures, which one has a nautical connection, so we’ve said which one has a connection to the sea. The piano accordion is one that we want to change, because it is not a commonly used thing here, and the harp. … if someone says it’s a musical instrument, then tick it off.”* (Julie)

### Interpreting and contextualising findings

Something that was stressed throughout the interviews was the importance of contextual information and clinician experience in conducting an assessment and interpreting the results.

*“It’s just really, really important to try and gather as much information as you can and to really - be prepared to just work with the complexity of it and not sort of jump to what would be a rather simplistic sort of interpretation of it, because there are so many factors which can influence these scores and so I guess there’s a responsibility on the part of the practitioner to make sure that they really are as well versed as they can be in the things that actually impact on these scores.”* (Belinda)

*“You’ve really got to have a wide source of information before [you] look at your interpretation. Because … if you’re getting a low score you’ve got to say, ‘Okay, why am I getting a low score here?’ and then look at all the other factors”.* (Sarah)

The key was identifying discrepancies and trying to account for them. For example, most would always do more than one assessment and look at the patterning of results. It wasn’t about the test score, but why they got that score and whether it fit the clinical profile expected for that person given their individual circumstances, history, language ability, literacy or education level.

*“If you’re looking at, let’s say, Korsakoff Syndrome… We know what that means, and that means that the working memory should remain relatively intact, where all the other numbers drop. So if you’re looking at something like Wernicke-Korsakoff’s and you find that, okay the working memory is high, but you’ve got two other ones that are also high, you start thinking, ‘No. There’s something else at work here’.”* (Sampson)

Clinicians would also look for concordance between different sources of information. In their interpretation they would assess how well the scores reflected their clinical impression of the person based on informal or observational data collection during the interview, as well as information from other sources such as talking with family or teachers, or reviewing medical history.

*“But that’s where you’ve got to triangulate by getting a bit here and a bit there and a bit there and comparing them and having a look… So it’s the sort of gestalt that you are looking at, this person and the history and you pull it all together …”* (Martin)

*“I would have to say the most effective method of assessing cognition is a combination of interview and observations. The tools that exist at the moment just don't weigh up enough compared to that. If you were to just run with a tool I think you'd be quite deceived.”* (David)

Clinicians would examine whether there was a medical reason for the result, for example hearing or vision problems, transient delirium or psychosis, medication or intoxication. Most would also take note of qualitative information during the testing process such as whether they were engaged, motivated, tired, distracted, hungry or actually understood what was being asked etc.:

*“It's about having a fair assessment. Say with block design it's timed, and we'll say ‘In 45 seconds they were half way through, they were keen to work for another three minutes and finish the design’. So you can't score it as a finished design but it's different from someone who didn't try and then 45 seconds are up.”* (Felicity)

*“Even the fact that sometimes they don’t understand what you’ve asked them to do is part of the assessment.”* (Kirsty)

The context in which the assessment was conducted is also important to consider and might impact (negatively or positively) upon the results.

*“I think the whole testing situation is problematic, in that it’s unfamiliar, and often it is out of country and somebody who’s not known in the service, all that sort of meta-cognitive understanding.”* (Neil)

*“Have you ever been to emergency in [the hospital]? It's not relaxing, they're obviously not well. Having said that, if they’ve been in for eight hours, for some people they’ve had a feed, they’ve had a sleep, so they're really well. They are better than they have been.”* (Felicity)

## Discussion

The results of this study highlighted a relative lack of consistency and guidance around appropriate cognitive testing methods for use with Aboriginal Australians in clinical practice. Practitioners often developed informal methods for assessing cognition or relied on mainstream standardised tests which they adapted or with which they were highly familiar in order to be able to assess the validity of the results. Some reported becoming “fond of” or developing a “love affair” with particular tests suggesting familiarity with the assessment was essential to be able to interpret mainstream tests in a culturally fair manner. Nevertheless, the importance of tailoring assessments to the individual was emphasised. Test selection, delivery and interpretation therefore relied heavily on experience and clinical judgement, leaving inexperienced clinicians with little guidance or confidence. While it appeared that clinical training did not equip emerging clinicians with the appropriate skills and knowledge to assess cognition confidently for Aboriginal clients, the Australian Psychological Society (APS) *Guidelines for the provision of Psychological Services for and the Conduct of Psychological research with Aboriginal and Torres Strait Islander People in Australia* were largely reflected through clinicians’ narratives [21].

As indicated by the APS guidelines, clinicians placed importance on having a sound understanding of the test, including its limits, and often noted caveats on test interpretation [21]. They also reflected the importance of i) having a good understanding of the reason for the assessment, ii) collecting detailed biographical and contextual information, and iii) combining test results with other forms of assessment, information and/or indicators of daily functioning. Implicit in clinician responses is an underlying distinction between ‘testing’ and ‘assessment’ that has been recognised previously [17,22]. In 1995, Davidson [17] proposed a multi-axial model of cognitive assessment for Aboriginal Australians, promoting assessment of cognitive functioning through examining individual functioning in particular contexts using measures of competence in performance of every-day life tasks; cognitive assessment through the use of appropriate neuropsychological and cognitive tasks; and developing contextual scales including complexity of the cognitive functions involved, the familiarity and perceived difficulty of the task, and levels of acculturation. The results of this study suggest that clinicians may ultimately be aligning with this type of model (see Figure 1).

Evidently, issues such as language, acculturation, literacy and education level were considered to impact the assessment process with time and relationship also identified as important factors [11]. Issues included a different concept of time between Aboriginal and non-Aboriginal people that has been described previously [10,23], and the impact that difference has on timed tasks, the amount of time taken to develop rapport and trust between the practitioner and Aboriginal client, the ability of a test or practitioner to hold a client’s attention and engage them long enough to complete a thorough assessment, and time constraints regarding different bureaucratic or practice settings. Taking sufficient time to develop rapport, trust and engagement emerged as an important theme in working with Aboriginal clients, reflecting previous findings in this setting [2]. Sheldon [2] provides detailed descriptions of appropriate and useful behaviours for engagement and, as in our study, techniques such as finding a comfortable place to sit, identifying common connections, humour, and being vouched for proved useful.

While tests themselves are just one component of an assessment that may be more or less valid for Aboriginal people, having a valid and fair interpretation of the test was considered vitally important. Caveats were almost always placed on test scores and their interpretations. While some guidance for clinicians on test selection might be useful, what could prove even more useful is guidance around achieving a fair interpretation of the results.

### Test selection

The range of assessments appropriate for Aboriginal clients is highly limited, bordering on negligent [4]. However, the use of standardised tests is often required for both bureaucratic and therapeutic reasons. A previous study observed poor concordance between counsellors’ clinical impression of cognitive functioning and neuropsychological test results, despite impressions being based on information sources including detailed history, clinical interview, medical examination and substance misuse history [3]. In this study, minimising reliance on assumptions, or purely clinical judgement, was considered important for achieving a reliable assessment. Assessments utilised most often included the KICA, Wechsler scales, Matrices tests such as the K-BIT, Raven’s Progressive Matrices, the TONI, and the NNAT in conjunction with observation, informal assessment and functional assessments.

Clinicians operated on a hypothesis-testing basis, founded on a thorough history and clinical impression. The functions expected to be impaired (or preserved) given the history (e.g. alcohol misuse), or the suspected profile of impairment (e.g. Wernicke-Korsakoff Syndrome) were important considerations in choosing an assessment, to ensure the relevant functions were covered. The level of acculturation, literacy, education and English language use were also important considerations, with non-verbal assessments relied upon heavily for individuals where English is a second language. Regardless, the concepts upon which tests are based are often unfamiliar to individuals from non-western cultures and consequently this must be considered in interpreting any results, and importantly, in designing more appropriate tests.

### Test interpretation

Gathering contextual information, informal assessments, indicators of daily functioning and triangulating these was seen as vitally important in interpreting any test. The impact of issues including the clinician/client relationship, language difficulties, whether the client’s basic needs are met, prolonged chronic trauma, poverty, and mistrust of mainstream systems are important considerations, particularly for Aboriginal clients. For most clinicians this relies heavily on extensive experience in Aboriginal settings and clinical judgement. It was therefore considered valuable to put some structure around the questions experienced clinicians ask in considering the validity of an Aboriginal assessment. Table 2 presents an ‘Interpretation Guide’ that was developed from clinician responses and subsequently refined by the participant reference group. It was considered important to stress that an assessment is only as good as what follows and that test scores should be interpreted in terms of the impact on the person’s daily functioning.

**Table 2:** Considerations for test interpretation in an Indigenous context

**Interpretation guide**	
**1.**	**Detailed History**
	**• Info from others**
	Have I talked to family, teachers, or others to find out about behaviour at home?
	○ Self care
	○ Social interaction or romantic relationships
	○ Memory, bizarre behaviour, any violation of cultural norms
	○ Activities of daily living
	○ Comparison to peers re language development & abilities
	○ Money management, knowledge of pension etc
	**• Medical History**
	Have I got a medical history from doctors, the client, family or medical files?
	○ Hearing and vision
	○ Medication use
	○ Previous head injury or other mental health or neurological condition
	**• Info from client**
	Have I got a social history from the client?
	○ Biographical information, family structure (e.g. genogram)
	○ Any personal or parental substance use (alcohol, tobacco, cannabis, petrol)
	○ Social circumstances, are their basic needs met (e.g. food, shelter, power, meds)?
	○ Schooling, work, jobs, or meaningful occupation
	○ Relationships/family functioning
	○ Legal issues
**2.**	**Testing Process**
	Were there any factors influencing the assessment process and what was the impact?
	**•** Context – could anything about the setting have impacted the results?
	○ Background noise, interruptions, clinician/client gender difference, away from homelands, chaotic setting, pain, discomfort, hunger, family worries, house worries, health worries?
	**•** Motivation – Were they motivated to do well? Do they distrust mainstream systems?
	**•** Engagement – Were they engaged in the process? Was the relationship affable & appropriate?
	**•** Tiredness/alertness – Did they sleep last night? Do they have a place to sleep?
	**•** Intoxication/medications? – Are they taking any drugs and/or do they *need* medications?
	**•** Perceptual issues – Hearing/vision. Do they need a hearing aid or glasses? Was there background noise/distractions?
	**•** Understanding – Did they understand what you asked them to do?
	**•** Language – Do they need an interpreter? Is the interpreter an appropriate person/relationship?
	**•** Item format – Timed?, question/answer?, pencil/paper?
**3.**	**Test Results**
	What is the pattern of scores?
	**•** Where are the discrepancies and is it what I would expect based on my hypotheses about the source of impairment?
**4.**	**Triangulating**
	Is there concordance between scores, clinical impression, daily functioning and other information?
	**•** If not, why not? Did the factors above impact?
**5.**	**Implications**
	All things considered, is this a reliable assessment?
	**•** If not, do I need more information and can I get it?
	**•** Is further assessment required?
	**•** What can I responsibly do with/say about the results?
	**•** How will this impact their everyday functioning?

### Limitations

The generalisability of these findings is limited as only clinicians working in the Northern Territory were recruited and only one was Aboriginal. In addition, the perspectives of other Aboriginal people in relation to appropriate cognitive assessment methods need to be further explored.

## Conclusions

Cognitive assessments developed specifically for Aboriginal people are urgently needed. Significant research effort is needed to develop and validate more appropriate assessments that are based on the skills and abilities taught and valued in Aboriginal cultures. In the absence of appropriate, validated assessments, clinicians have relied on a range of standardised and informal assessments whilst recognising the severe limitations of these. The reliability and validity of standardised tests or informal tasks for use with Aboriginal people is often not known. Clinicians must therefore be very cautious in their interpretation and use and rely on many other sources of information to inform the overall clinical picture. Past clinical training has not prepared clinicians adequately for assessing Aboriginal clients and instead expertise tends to develop over many years of experience, with significant trial and error. Practitioners with much experience (5-20+ years) in assessing cognitive function for Aboriginal people have developed a wealth of practical approaches to achieving fair assessments for their Aboriginal clients. While this study attempts to capture these learnings in a way that can serve as a resource for others, further work is needed to develop additional resources and to expand their reach and utility. Such work might include development of an online repository for collating and sharing knowledge from practitioners experienced in the field, a greater emphasis on teaching appropriate methods during education and training and establishing support mechanisms such as mentoring and ongoing professional development.

## Abbreviations

APS: Australian Psychological Society; IQ: Intelligence Quotient; KBIT: Kaufman’s Brief Intelligence Test; KICA: Kimberley Indigenous Cognitive Assessment; Menzies: Menzies School of Health Research; NNAT: Naglieri Non-verbal Ability Test; NT: Northern Territory; OT: Occupational Therapist; TONI: Test of Non-verbal Intelligence; WAIS: Wechsler Adult Intelligence Scale; WISC: Wechsler Intelligence Scale for Children; WMS: Wechsler Memory Scale; WNV: Wechsler Non-Verbal.

## Competing interests

The authors declare that they have no competing interests

## Authors’ contributions

KD conceived of and designed the study, carried out data collection, analysis and interpretation and prepared the draft manuscript. JP and ML contributed to the research plan, data collection and analysis and critically revised the draft manuscript. All authors read and approved the final manuscript.

## Pre-publication history

The pre-publication history for this paper can be accessed here:

http://www.biomedcentral.com/1471-244X/13/42/prepub
